# A comparison of resveratrol and other polyphenolic compounds on Notch activation and endothelial cell activity

**DOI:** 10.1371/journal.pone.0210607

**Published:** 2019-01-17

**Authors:** Bryce LaFoya, Jordan A. Munroe, Allan R. Albig

**Affiliations:** 1 Biomolecular Sciences PhD Program, Boise State University, Boise, Idaho, United States of America; 2 Department of Biological Sciences, Boise State University, Boise, Idaho, United States of America; East Carolina University, UNITED STATES

## Abstract

Resveratrol is a polyphenolic compound produced by plants which makes its way into the human diet through plant-based foods. It has been shown to provide many health benefits, helping to ward of age-related diseases and promoting cardiovascular health. Additionally, resveratrol is a potent activator of the Notch signaling pathway. While resveratrol receives the most attention as a polyphenolic nutraceutical, other compounds with similar structures may be more potent regulators of specific cellular processes. Here, we compare resveratrol, apigenin, chrysin, genistein, luteolin, myricetin, piceatannol, pterostilbene, and quercetin for their ability to regulate Notch signaling. In addition, we compare the ability of these polyphenolic compounds to regulate endothelial cell viability, proliferation, and migration. Out of these compounds we found that resveratrol is the best activator of Notch signaling, however, other similar compounds are also capable of stimulating Notch. We also discovered that several of these polyphenols were able to inhibit endothelial cell proliferation. Finally, we found that many of these polyphenols are potent inhibitors of endothelial migration during wound healing assays. These findings provide the first side-by-side comparison of the regulation of Notch signaling, and endothelial cell proliferation and migration, by nine polyphenolic compounds.

## Introduction

Our understanding of the role in which diet shapes human health is constantly evolving. A nutraceutical is a compound found naturally in food which has medicinal benefits. The use of nutraceuticals to combat disease and improve health is an ever-expanding area of research. One class of molecules, known as polyphenols, are derived from various plants and are renowned for their health benefits. Major sources of dietary polyphenols include tea, wine, coffee, chocolate, vegetables, and beer [1]. However, the molecular mechanisms by which these polyphenolic compounds affect human health are unclear.

Perhaps the best-studied polyphenol, trans-resveratrol (RSVT), has been characterized for its anti-aging [2], anti-cancer [3,4], anti-oxidant [5], anti-inflammatory [6], and neuroprotective [7–9] properties. RSVT is a polyphenolic stilbene derived from plants, such as grapes and peanuts [10]. In plants, it acts as a phytoalexin, protecting plant tissues against pathogenic assault [11]. Once ingested by humans, RSVT is thought to promote many favorable physiological processes such as the maintenance of vascular health, prevention of atherosclerosis [12,13], inhibition of tumor angiogenesis [14–18], and improvement of cardiovascular function [19–21]. While RSVT receives the most attention, many other polyphenols have been found to have similar activities to RSVT. There exists a vast literature describing the molecular mechanisms by which RSVT governs endothelial cell behavior, but little is known about how other polyphenols perform similar roles.

RSVT, has been heavily-linked with the Notch cell signaling pathway [22–24]. Despite the clear association between RSVT and Notch, conflicting results from different cell lines suggest that RSVT can enhance [23,25] or suppress [26] Notch in a cell type dependent manner. Being a form of juxtacrine cell communication, Notch signaling begins when the transmembrane Notch receptor of one cell (i.e. signal receiving cell) is bound by a transmembrane ligand on an adjacent cell (i.e. signal sending cell). A force of 4–12 pN [27] is applied to the Notch receptor through ligand endocytosis in the signal sending cell. This pulling force exposes cleavage sites and facilitates proteolytic processing of the Notch receptor, first by ADAM (A Disintegrin and Metalloproteinase) and then by γ-secretase [28]. These cleavage events result in the release of the Notch intracellular domain (NICD), which then travels to the nucleus where it induces transcription of Notch target genes. Hairy and enhancer of split (HES) genes and hairy/enhancer of split related with TYRPW motif (HEY) genes are well-known examples of Notch target genes [29].

Here, we compare RSVT and several other polyphenols for their ability to regulate Notch signaling and endothelial cell proliferation and migration. We chose to compare the effects of RSVT with apigenin, chrysin, genistein, luteolin, myricetin, piceatannol, pterostilbene, and quercetin in order to see if these molecules, which share similar structures, behave similarly to one another. We found that the majority of these polyphenols, but not all, enhanced Notch signaling to varying degrees. Similarly, the majority of tested polyphenols, but not all, inhibited cell proliferation and migration. These results should prove useful to other researchers seeking to harness the biochemical properties of polyphenols for therapeutic uses.

## Materials and methods

### Cell culture

293T cells were cultured in Dulbecco’s Modified Eagle’s Medium (DMEM, Mediatech) supplemented with 10% fetal bovine serum (FBS) and 1x pen-strep. Human Aortic Vascular Smooth Muscle Cells (HAVSMC) were cultured in EBM2 basal media (Lonza) supplemented with EGM2 growth media and 10% FBS. Human Microvascular Endothelial Cells (HMEC-1) were cultured in MCDB131 supplemented with 10% FBS, 10 ng/ml epidermal growth factor, and 1 μg/ml hydrocortisone. Cells were grown in 10 cm plates and passaged before reaching confluency.

### Materials

Trans-RSVT was purchased from Caymen Chemicals. Apigenin, chrysin, luteolin, and quercetin were purchased from Alfa Aesar. Myricetin, piceatannol, and pterostilbene were purchased from Enzo Life Sciences. Genistein and doxycycline were purchased from Tokyo Chemical Industry. All drugs were dissolved in DMSO.

### Plasmids

The N1ICD construct (Addgene #20183) was a gift from Raphael Kopan and contains amino acids Val1744 to Lys2531 of the mouse Notch1 intracellular domain with a 3xFLAG N-terminal tag [30]. N1ICD was inserted into a doxycycline inducible lenti viral destination vector, pCW57.1 (Addgene #41393, a gift from David Root) in order to construct a N1ICD lenti viral expression vector. The 4xCSL luciferase construct (Addgene #41726) was a gift from Raphael Kopan and contains 4 tandem repeats of the high affinity CSL binding sites (5′CGTGGGAA3′) while transcribing for firefly luciferase [30]. The Hes1 luciferase construct was a gift from Jan Jensen and consists of nucleotides -2553 to -201 relative to the murine Hes1 transcriptional start site while transcribing for firefly luciferase. The Hes5 luciferase construct (Addgene #41724) was a gift from Ryoichiro Kageyama and Raphael Kopan and contains the murine Hes5 promoter (-800 to +73) relative to the Hes5 transcriptional start site while transcribing for firefly luciferase [31].

### Apoptosis assays

HMEC-1 cells were seeded into 6 well plates at a density of 150,000 cells/well and allowed to grow for 24 hours. Cells were then treated with 0–100 μM concentrations of RSVT and/or 10 μM of the Notch inhibitor DAPT, and allowed to incubate for 24 hours. After incubation, cell culture media was collected and cells were lysed in SDS page lysis buffer. Cell culture media was pelleted and added to cell lysates. For a positive control for apoptosis, cells were exposed to 15 minutes of ultraviolet (UV) light before lysing. Apoptosis was monitored through western blotting for the presence of the apoptotic marker, cleaved caspase 3.

### Luciferase assays

HMEC-1 and HAVSMC cells were seeded into 24-well plates at a density of 25,000 cells/well. 293T cells were seeded into 24-well plates at a density of 50,000 cells/well. The following day, cells were transfected using LT-1 liposomes (Mirus). Cells were transfected with 100 ng/well Hes1 luc, Hes5 luc, or 4xCSL luc plasmids which produce luciferase in response to Notch pathway activation. Co-transfection with 30 ng/well of a CMV-Beta-Galactosidase construct was used to normalize data for transfection efficiency and potential cell death/proliferation. Cells were lysed 48 hours after transfection using passive lysis buffer (Promega) and lysates were used to perform a luciferase reporter assay as per manufacturer’s protocol and analyzed using a Promega Glomax Multi Detection System luminometer. Luciferase activity was normalized to Beta-Galactosidase activity and values were reported as fold change to control. All conditions were performed in triplicate for each independent experiment.

### Cell viability assays

HMEC-1 cells were seeded into 96-well plates. Upon reaching confluency, cells were treated with 0, 1, 10, and 100 μM polyphenols. After 24 hours, a triplicate of wells for each condition was analyzed for cell viability using a WST-1 colorimetric assay. Absorbance spectra was measured at 410 nm using a BioTek Synergy Mx plate reader. Quantification of cell viability was reported as a percentage of DMSO control (0 μM polyphenol).

### Proliferation assays

HMEC-1 cells were lenti viral transduced with doxycycline inducible constructs that contain WT N1ICD under the control of a CMV promoter. Cells were treated with 10 μM polyphenols and seeded into 96-well plates at a density of 2,500 cells/well. Doxycycline was added to appropriate wells in order to induce N1ICD overexpression. After 24, 48, and 72 hours, a triplicate of wells for each condition was analyzed for cell density using a WST-1 colorimetric assay. Absorbance spectra was measured at 410 nm using a BioTek Synergy Mx plate reader.

### Scratch assays

HMEC-1 cells were seeded into 24-well plates at a density of 25,000 cells/well. Upon reaching confluency cells were treated with 10 μM and incubated for 24 hours. Wounds were made using 200 μL pipette tips. Wells were washed 3 times with 1x PBS and media/polyphenol treatments were replaced. Cells were placed in on on-stage incubator (5% CO_2_, 37°C). Images were captured using an EVOS FL auto microscope which automatically captured images every 30 minutes for 18 hours. After 18 hours, images were analyzed and wound area was calculated using ImageJ. Percent area of wound closure was calculated using the following formula, (area of wound at 0h - area of wound at 18h) / (area of wound at 0h X 100).

### Western blotting

Cells were lysed in 1x SDS page lysis buffer and boiled for 5 minutes. Proteins were separated through SDS page on 6%-15% polyacrylamide gels and blotted onto nitrocellulose membranes. Membranes were blocked in TBS-T (140 mM NaCL, 25 mM Tris-HCL, pH 7.4, 0.1% Tween-20) with 5% bovine serum albumin for 1 hour at room temperature. Membranes were incubated with primary antibody (1:250, 1:500, or 1:1000) overnight on a rotator at 4°C. After incubation, membranes were washed 3 x 10 minutes in TBS-T before 1 hour incubation in secondary antibodies at room temperature. Horseradish peroxidase conjugated secondary antibodies were used at a concentration of 1:5000. After incubation with secondary antibodies, proteins were detected by enhanced chemiluminescence. Primary antibodies against β-actin (sc-47778) were purchased from Santa Cruz Biotechnology. Primary antibodies against caspase 3 (#9662) were purchased from Cell Signaling Technology.

## Results and discussion

### RSVT induces Notch target gene transcription

**Fig 1:**
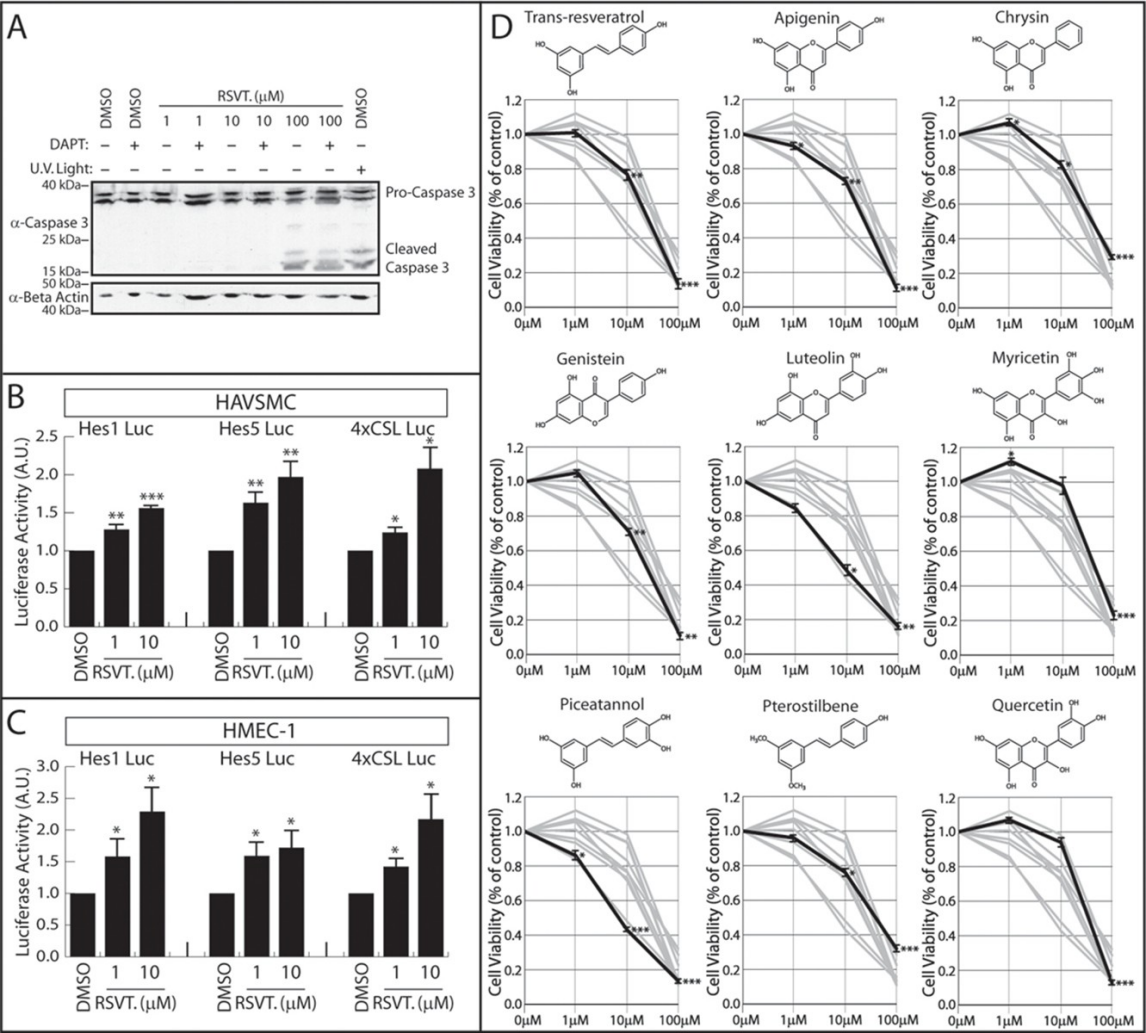
RSVT is a potent stimulator of Notch signaling. (A) RSVT does not induce HMEC apoptosis at 1–10 μM. HMEC cells were treated with increasing concentrations of RSVT +/- DAPT. Cellular apoptosis was indirectly examined by monitoring cleavage of cleaved caspase 3 from pro-caspase 3 by western blot. HMEC cells were treated with UV light as a positive apoptosis control and protein loading was monitored by western blotting for α-actin. Data shows that RSVT induces apoptosis at 100 μM. (B) Notch activation measured by three luciferase reporter constructs in HAVSMC cells. Student’s t-test was performed to determine statistical significance compared to 0 μM RSVT control. P-values are reported as * < .05, ** < .01, *** < .001. Data represents n≥5. Data shows that RSVT induces Notch target gene transcription in HAVSMC cells. (C) Notch activation measured by three luciferase reporter constructs in HMEC-1 cells. Student’s t-test was performed to determine statistical significance compared to 0 μM RSVT control. P-values are reported as * < .05, ** < .01, *** < .001. Data represents n≥5. Data shows that RSVT induces Notch target gene transcription in HMEC-1 cells. (D) Cell viability of HMEC-1 cells measured using WST-1 cell viability assay. Cell viability was quantified 24 hours after treatment with nine different polyphenolic compounds. Cell viability as a percentage of DMSO control (0 μM) is graphed. Data represents the average of three replicate experiments. Structures of each polyphenol are shown above respective graph. Bolded line depicts cell viability measured for each polyphenol. Student’s t-test was performed to determine statistical significance compared to DMSO control. P-values are reported as * < .05, ** < .01, *** < .001. Data demonstrates that most polyphenols did not drastically compromise cell viability when used at 1 μM and 10 μM concentrations. However, 100 μM polyphenol concentrations significantly reduced cell viability for all nine polyphenols tested.

A robust literature exists connecting RSVT with the Notch signaling pathway. The association between Notch and RSVT was first established when Pinchot et al. employed a high throughput chemical screening method to screen 7,264 compounds in order to identify Notch activating compounds [22]. Out of all the compounds screened in this study, RSVT was identified as the strongest Notch activator. RSVT has been shown to induce apoptosis of endothelial cells [32,33] and it was therefore important to first determine a sub-apoptotic concentration of RSVT in which to examine Notch activation. Human Microvascular Endothelial Cells (HMEC-1) were cultured in 1 (.23 μg/ml), 10 (2.3 μg/ml), or 100 (23 μg/ml) μM concentrations of RSVT in the presence or absence of the Notch inhibitor DAPT and apoptosis was monitored by western blot analysis of the apoptosis marker, cleaved caspase 3 (Fig 1A). Similar to previous studies [32], we found that 100 μM solutions of RSVT induced caspase 3 cleavage, but 1–10 μM RSVT showed no evidence of apoptosis. Notch inhibition did not induce apoptosis at 0–10 μM RSVT concentration, and did not reverse or enhance the stimulation of apoptosis by 100 μM RSVT treatments. To determine the effect of RSVT on Notch signaling, we transfected HMEC-1 cells and Human Aortic Vascular Smooth Muscle Cells (HAVSMC) with Notch responsive Hes1, Hes5, and 4xCSL luciferase constructs and incubated these cells in the presence of 1–10 μM RSVT. In both cell types, and across all three Notch-responsive reporters, RSVT activated Notch target gene transcription in a dose-dependent manner (Fig 1B and 1C). These results demonstrated that RSVT controls Notch independent of apoptosis and established a model on which we could examine additional polyphenols for Notch regulatory activity.

### Polyphenols and endothelial cell viability

Having studied the effects of RSVT on apoptosis, we next compared cell viability upon treatment with eight other polyphenolic compounds which share similar structures to RSVT. Apigenin, chrysin, genistein, luteolin, myricetin, piceatannol, pterostilbene, and quercetin were chosen based on their structural similarity to RSVT, compared to other polyphenols such as epigallocatechin gallate (EGCG) or curcumin which are structurally less similar to RSVT. We performed cell viability assays in HMEC-1 cells grown to confluency, then treated for 24 hours with various concentrations of nine polyphenols (Fig 1D). With the exception of luteolin and piceatannol, low concentrations (1 μM and 10 μM) of polyphenols did not significantly reduce cell viability. For all the polyphenols tested, 100 μM concentrations significantly reduced cell viability.

### Other polyphenols induce Notch target gene transcription

**Fig 2:**
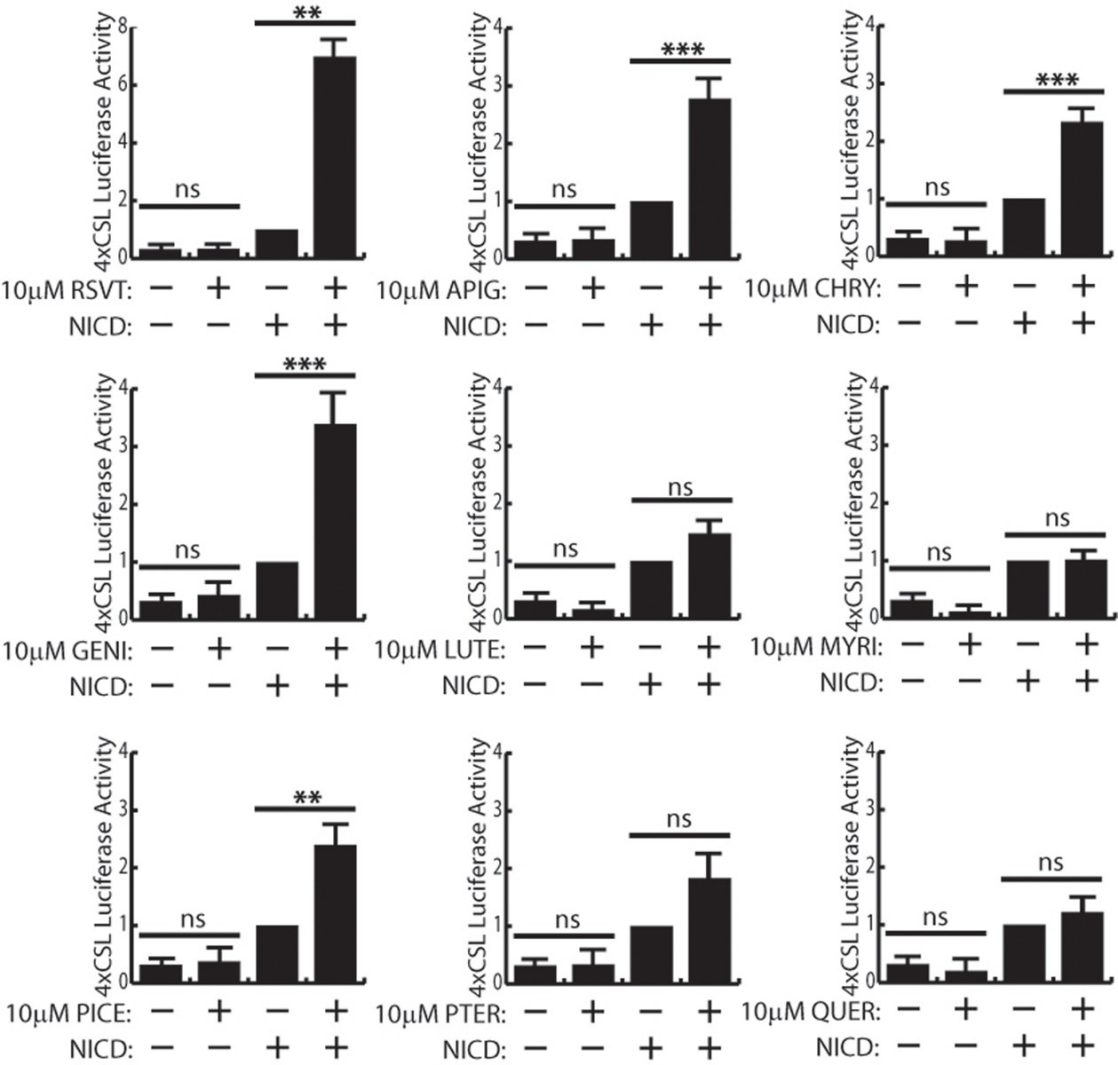
Other polyphenolic compounds are Notch activators. Notch activation measured by 4xCSL luciferase assays in 293T cells, by nine different polyphenols compared to DMSO (-) control, in the presence (+) or absence of N1ICD (-) overexpression. Student’s t-test was performed to determine statistical significance. P-values are reported as * < .05, ** < .01, *** < .001. Data represents n≥5. Data shows that resveratrol (RSVT), apigenin (APIG), chrysin (CHRY), genistein (GENI), and piceatannol (PICE) activate Notch target gene transcription, but only in the presence of NICD. Luteolin (LUTE), myricetin (MYRI), pterostilbene (PTER), and quercetin (QUER) do not activate Notch target gene transcription.

Having confirmed RSVT activation of Notch signaling, we next made a direct comparison of Notch induction by eight other polyphenols. In this assay, 293T cells were used because they are easily transfectable compared to HMEC-1 and HAVSMC cells. Based on our cell viability assays, we employed a 24 hour treatment of 10 μM polyphenols to study polyphenolic regulation of Notch activity. We transfected 293T cells with the 4xCSL luciferase reporter and compared luciferase signal in the absence or presence of various polyphenols. Since 293T cells exhibit very low endogenous Notch activity, none of the polyphenols we tested demonstrated any effect on endogenous Notch activity (compared to DMSO control) in these cells (Fig 2). Therefore, we also examined the effect of polyphenols on Notch signaling in 293T cells that were transfected with cDNA encoding the Notch1 intracellular domain (N1ICD) to activate Notch. N1ICD co-transfection enhanced basal 4xCSL promoter activity, and treatment with polyphenols elicited a variety of results (Fig 2). As demonstrated in HMEC-1 and HAVSMC cells, RSVT enhances Notch signaling in 293T cells also. Of all the polyphenols we tested, RSVT was the best inducer of Notch target gene transcription with a ~7-fold induction of luciferase activity over DMSO/+N1ICD control. Apigenin, chrysin, genistein, and piceatannol were also demonstrated to potentiate Notch target gene transcription to varying degrees less than RSVT. Luteolin, myricetin, pterostilbene, and quercetin did not activate or repress Notch transcriptional activity. This result demonstrated that although these polyphenols have similar chemistries, there are subtle and significant differences in their ability to activate Notch signaling.

### Polyphenolic regulation of endothelial cell proliferation

**Fig 3:**
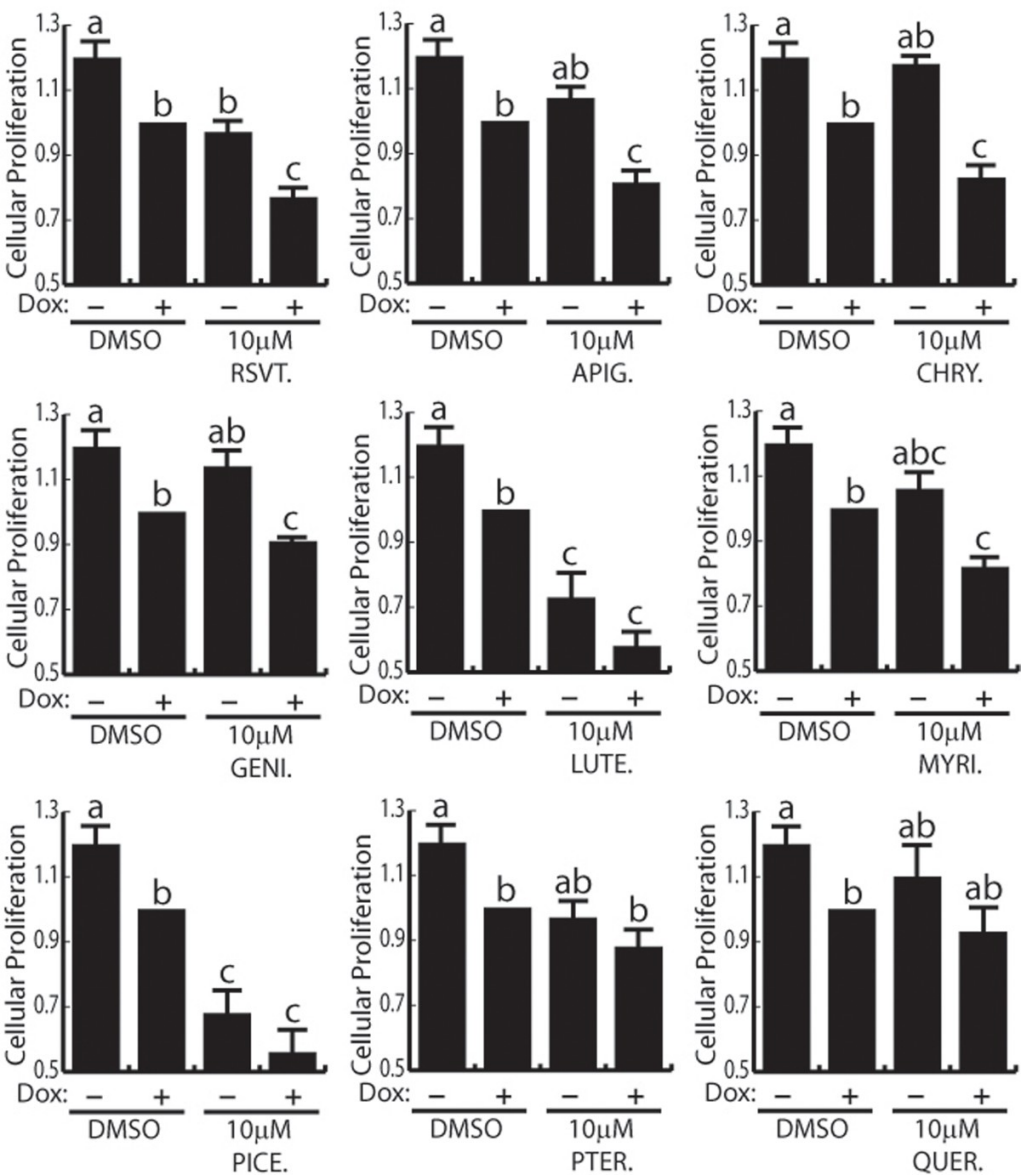
Polyphenolic regulation of endothelial cell proliferation. Proliferation of HMEC-1 cells measured by WST-1 proliferation assays. HMEC-1 cells which had been transduced with lentiviral particles encoding N1ICD under the control of a doxycycline inducible promoter were used. One-way ANOVA followed by Bonferroni’s post-hoc tests was performed to determine statistical significance. Differing letters represent statistical significant differences. Data represents n = 6. Polyphenols which inhibit HMEC-1 proliferation in a Notch dependent manner include RSVT, APIG, CHRY, MYRI and GENI. LUTE and PICE inhibit HMEC-1 proliferation during both basal and high Notch activity. PTER and QUER do not alter HMEC-1 proliferation.

There are conflicting reports on the role RSVT plays in the regulation of cellular proliferation [4,34] and there has not been a head-to-head comparison of polyphenol effects on endothelial cell proliferation. Given our results that many polyphenols control Notch signaling, we next compared the effect of RSVT and the other polyphenols on endothelial cell proliferation in the presence or absence of elevated Notch signaling. For this study we used HMEC-1 cells which had been transduced with lentiviral particles encoding N1ICD under the control of a doxycycline inducible promoter (HMEC-1-N1ICD cells). Subconfluent HMEC-1-N1ICD cells were treated with 10 μM polyphenolic compounds in the presence (i.e. high Notch activity) or absence (i.e. basal Notch activity) of doxycycline and cell proliferation was monitored daily by WST-1 over the course of 72 hours (Fig 3). In agreement with previous reports, overexpression of N1ICD reduced cell proliferation [35,36]. Under conditions of basal Notch activity (i.e. no doxycycline induction) only luteolin, and piceatannol were capable of inhibiting cellular proliferation. However, in the presence of elevated Notch activity, all of the polyphenols, except pterostilbene and quercetin, reduced cellular proliferation compared to DMSO control under conditions of high Notch activity. Overall, RSVT reduced cellular proliferation by 22%, but luteolin and piceatannol were the best inhibitors of proliferation, displaying a 42% and 46% reduction in cellular proliferation respectively. From this evidence, we conclude Notch activity is required for the inhibition of proliferation by RSVT, apigenin, chrysin, genistein, and myricetin. Whereas, high Notch activity potentiates, but is not required for the inhibition of proliferation by luteolin, and piceatannol. Finally, pterostilbene and quercetin did not significantly affect proliferation in our model system.

### Polyphenolic regulation of endothelial wound healing

**Fig 4:**
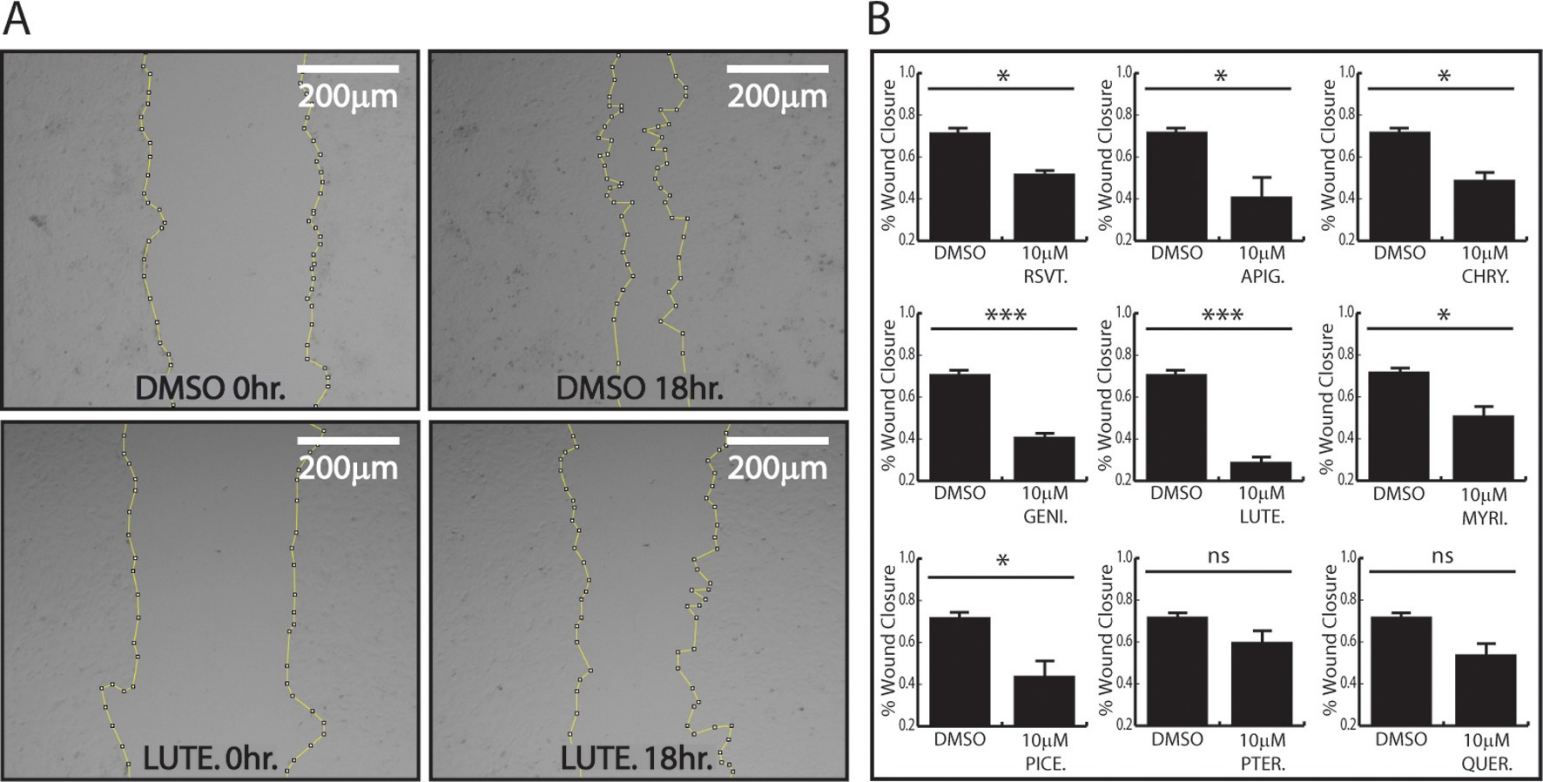
Polyphenolic regulation of endothelial cell migration. Migration of endothelial cells measured through scratch assay analysis. (A) HMEC-1 cells were grown in 10 μM luteolin or DMSO control for 24 hours prior to wounding. Micrograph images where taken at 0hrs and 18hrs after treatment. Area of wound is outlined. (B) HMEC-1 cells were grown to confluency and treated with 10 μM polyphenols or DMSO control for 24 hours prior to wounding. Data depicts % wound closure after 18 hours. Student’s t-test was performed to determine statistical significance. RSVT, APIG, CHRY, GENI, LUTE, MYRI, and PICE inhibit HMEC-1 cell migration, whereas PTER and QUER do not alter migration. P-values are reported as * < .05, ** < .01, *** < .001. Data represents n = 3.

RSVT and several other polyphenols have been shown to decrease endothelial migration, but a head-to-head comparison of how these polyphenols affect cell migration has not been reported. Therefore, we sought to compare the effect of various polyphenols on endothelial cell migration using a wound closure scratch assay. To this end, HMEC-1 cells were grown to confluency and treated with 10 μM concentrations of various polyphenols for 24 hours prior to monolayer wounding. After wounding, cells were allotted 18 hours for migration, followed by subsequent wound healing quantification as a percent area of wound closure. As previously observed [37], we found that RSVT significantly decreased endothelial cell migration (Fig 4B). We also found that apigenin, chrysin, genistein, luteolin, myricetin, and piceatannol also significantly inhibited endothelial cell migration (Fig 4B & Supplemental). Of these compounds, luteolin had the largest effect on migration, with only 30% wound closure after 18 hours (Fig 4A & 4B). Pterostilbene and quercetin did not reduce cell migration in a statistically significant manner.

## Conclusions

Notch signaling, endothelial cell proliferation, and endothelial cell migration are collectively important for angiogenesis and many polyphenolic compounds have been identified as regulators of angiogenesis. However, there has been no direct comparison of polyphenols on these cellular activities. Therefore, the goal of this study was to compare several polyphenolic compounds for their ability to control Notch signaling, endothelial cell proliferation, and endothelial cell migration. Throughout this study, we performed several side-by-side comparisons of the biological potency of nine polyphenolic compounds. Despite the highly similar structures of these polyphenols, we have found that some, but not all, of these natural products are activators of Notch signaling or inhibitors of endothelial cell proliferation and migration. Two of the polyphenolics (pterostilbene and quercetin) failed to show biological activity in any of the experimental systems we examined, except that they reduce cell viability at high concentrations.

Polyphenolic compounds fall into several categories according to their structure [38]. In this work, we examined several polyphenolics with similar structures including the stilbenes RSVT, piceatannol, and pterostilbene and the flavonoids apigenin, chrysin, genistein, luteolin, myricetin, and quercetin. Many polyphenolic compounds have been shown to control Notch signaling, however a direct comparison of the Notch regulating activities of these compounds has not been performed. Compared to other polyphenols, RSVT has received the most attention for its role in regulating the Notch cell signaling pathway [23,24]. Through our analysis, it is clear that RSVT warrants its attention as a robust polyphenolic activator of Notch as it demonstrated the greatest Notch inducing activity. While RSVT was the most potent Notch activator out of the polyphenols we tested, apigenin, chrysin, genistein, and piceatannol were also able to regulate Notch to lesser degrees. Our results are consistent with previous findings showing that chrysin [39], and genistein [40] can control Notch, however the findings that apigenin and piceatannol can also control Notch is novel. In contrast, two other polyphenols which have been previously identified as Notch regulators, luteolin [41,42] and pterostilbene [43], did not act as Notch regulators in the cell types we tested. Finally, myricetin and quercetin have not been linked to Notch activity, and our data does not support a Notch regulatory role for these polyphenols. Taken together, our results show that polyphenolic compounds are a promising source of Notch regulators, but also provide a warning that cell-type specific responses to polyphenols may account for conflicting data concerning these molecules.

While we found that RSVT works synergistically with Notch signaling to suppress endothelial cell proliferation, apigenin, chrysin, genistein, luteolin, myricetin, and piceatannol also demonstrated similar activity. In fact, out of the compounds we tested, luteolin and piceatannol were the most potent inhibitors of endothelial cell proliferation. Since luteolin and piceatannol have previously been identified as an anti-angiogenic agents [44,45], our identification of these compounds as suppressors of endothelial cell proliferation may provide mechanistic insight into their anti-angiogenic properties. Since Notch is known to induce endothelial cell senescence [36,46], and we found that many of these polyphenols only acted as anti-proliferative agents under conditions of high Notch activity, we speculate that these anti-proliferative effects may be Notch-dependent. Additionally, the effects these molecules have on cellular proliferation may expand beyond endothelium. Future work should assess the effectiveness of these polyphenols for their ability to suppress tumor growth. Previous work has found that luteolin [41,47] suppresses cell migration. In accordance, luteolin was the most potent inhibitor of endothelial migration we tested. Notch activation has also been shown to inhibit endothelial cell migration [48]. Additionally, genes working downstream of Notch activation, such as Hey1, are known to inhibit endothelial migration [49]. Thus, it is possible that polyphenolic-based stimulation of Notch signaling may be responsible for the anti-migratory effects we observed. While our identification of these molecules as inhibitors of endothelial migration is relevant to angiogenesis, future work should compare polyphenolic regulation of cancer cell migration for their potential use as anti-metastatic agents. The observed polyphenolic-based control over the endothelial cell behavior could be the result of crosstalk between Notch and other signaling pathways. Integrin β3 is known to bear a RSVT receptor site [50], and has been shown to be required for RSVT’s anti-angiogenic properties [14]. Our previous work has shown that integrin β3 acts as a Notch regulator [51,52]. Based on this, it is tempting to speculate that polyphenols modulate a Notch-integrin crosstalk mechanism to control endothelial proliferation and migration. However, more work would be needed in order to elucidate such a mechanism.

These results provide the first side-by-side comparison of nine polyphenolic compounds in their ability to regulate Notch signaling, and endothelial cell proliferation and migration. Angiogenic growth requires tight coordination of Notch signaling, endothelial cell proliferation, and migration in endothelial cells [53]. This study has demonstrated that polyphenols act as modulators of angiogenic processes (Summarized in Table 1). Future work should expand upon this analysis, comparing how polyphenols behave in more sophisticated angiogenic models. Additionally, while angiogenesis is an essential step in tumor progression, performing a comparative analysis of polyphenolic treatment in the context of cancer cell behavior is necessary, and would complement this endothelial-based study, in order to gain a better scope of the use of polyphenols as anti-cancer agents. More broadly, our findings have laid the groundwork for the potential use of polyphenols as modulators of any developmental or disease process in which Notch signaling, cellular proliferation, and/or cellular migration are involved. While further exploration is necessary, we have shown that polyphenols are promising anti-angiogenic compounds which may facilitate natural product based cancer-related therapies in the future.

**Table 1:** Summary.

	Notch activity	Endothelial cell proliferation	Endothelial cell migration
**Resveratrol**	Enhancer	Inhibitor	Inhibitor
**Apigenin**	Enhancer	Inhibitor	Inhibitor
**Chrysin**	Enhancer	Inhibitor	Inhibitor
**Genistein**	Enhancer	Inhibitor	Inhibitor
**Luteolin**	Neutral	Inhibitor	Inhibitor
**Myricetin**	Neutral	Inhibitor	Inhibitor
**Piceatannol**	Enhancer	Inhibitor	Inhibitor
**Pterostilbene**	Neutral	Neutral	Neutral
**Quercetin**	Neutral	Neutral	Neutral

The summarization of the ability of individual polyphenols to regulate Notch activity, endothelial cell proliferation, and endothelial cell migration.

## Supporting information

S1 Fig Scratch assays. Migration of endothelial cells measured through scratch assay analysis. HMEC-1 cells were grown to confluency and treated with 10 μM polyphenols or DMSO control for 24 hours prior to wounding. Micrograph images were taken at 0 hours and 18 hours after wounding. Area of wound is outlined.
(TIF)
