# Supplementary figures and images for: A comparison of resveratrol and other polyphenolic compounds on Notch activation and endothelial cell activity
Source: PLoS One. 2019 Jan 17;14(1):e0210607. doi: 10.1371/journal.pone.0210607 (PMC6336259; doi:10.1371/journal.pone.0210607)

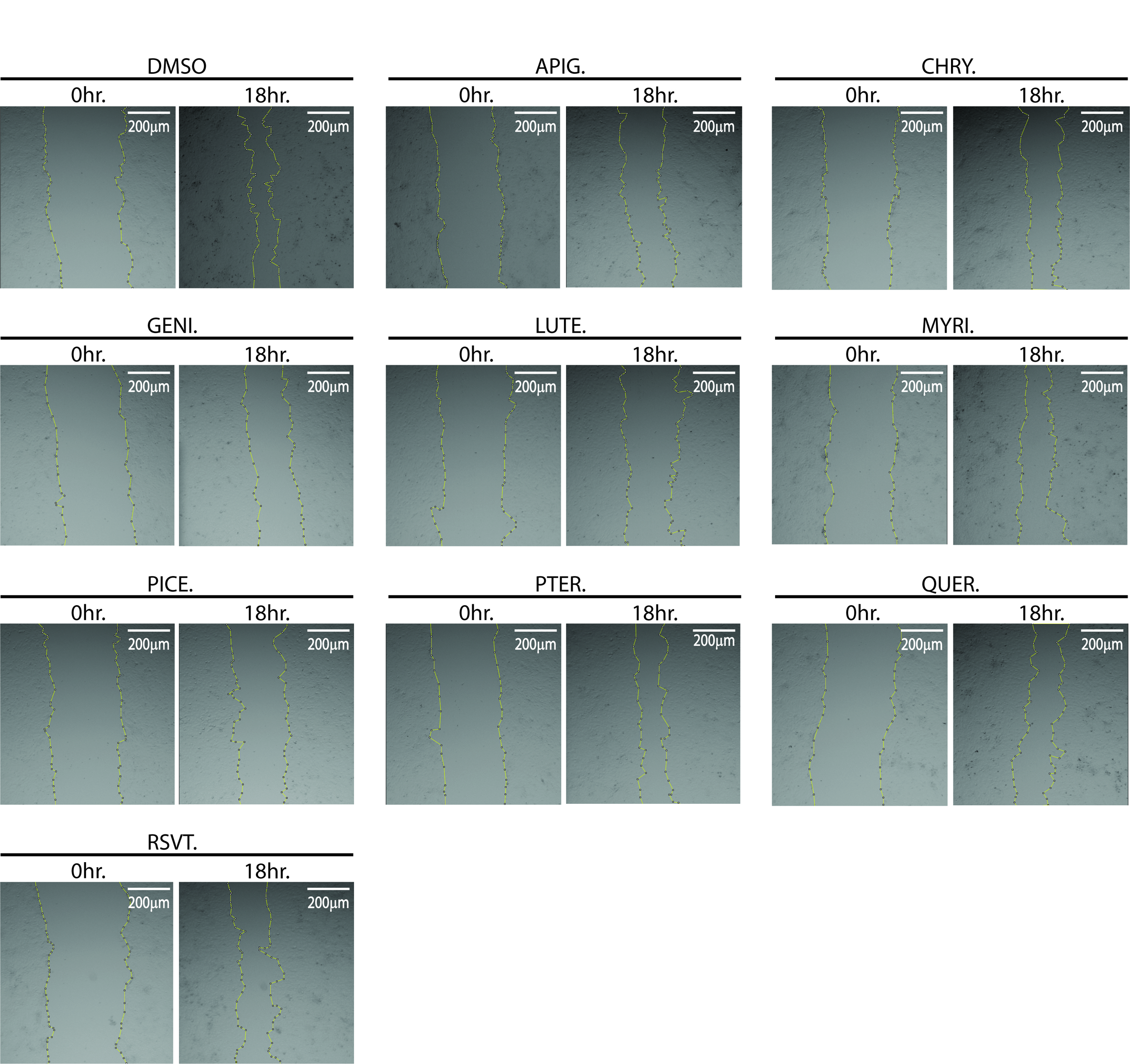

Supplement: S1 Fig — Migration of endothelial cells measured through scratch assay analysis. HMEC-1 cells were grown to confluency and treated with 10 μM polyphenols or DMSO control for 24 hours prior to wounding. Micrograph images were taken at 0 hours and 18 hours after wounding. Area of wound is outlined. (TIF) [file pone.0210607.s001.tif]
